# Seasonality and brain size are negatively associated in frogs: evidence for the expensive brain framework

**DOI:** 10.1038/s41598-017-16921-1

**Published:** 2017-11-30

**Authors:** Yi Luo, Mao Jun Zhong, Yan Huang, Feng Li, Wen Bo Liao, Alexander Kotrschal

**Affiliations:** 1 Key Laboratory of Southwest China Wildlife Resources Conservation (Ministry of Education), China West Normal University, Nanchong, 637009 Sichuan China; 2 Institute of Eco-adaptation in Amphibians and Reptiles, China West Normal University, Nanchong, 637009 Sichuan China; 3 Zoological Institute, Stockholm University, 10691 Stockholm, Sweden

## Abstract

The challenges of seasonal environments are thought to contribute to brain evolution, but in which way is debated. According to the Cognitive Buffer Hypothesis (CBH) brain size should increase with seasonality, as the cognitive benefits of a larger brain should help overcoming periods of food scarcity via, for instance, increased behavioral flexibility. However, in line with the Expensive Brain Framework (EBF) brain size should decrease with seasonality because a smaller brain confers energetic benefits in periods of food scarcity. Empirical evidence is inconclusive and mostly limited to homoeothermic animals. Here we used phylogenetic comparative analyses to test the impact of seasonality on brain evolution across 30 species of anurans (frogs) experiencing a wide range of temperature and precipitation. Our results support the EBF because relative brain size and the size of the optic tectum were negatively correlated with variability in temperature. In contrast, we found no association between the variability in precipitation and the length of the dry season with either brain size or the sizes of other major brain regions. We suggest that seasonality-induced food scarcity resulting from higher variability in temperature constrains brain size evolution in anurans. Less seasonal environments may therefore facilitate the evolution of larger brains in poikilothermic animals.

## Introduction

Brain size varies dramatically between species^1^ and the recent decades have uncovered a variety of factors that drive this evolution^2–10^. Consequently, several adaptive hypotheses have been proposed to explain the evolution of brain size in vertebrates. Most of these hypotheses assume that due to its cognitive benefits a larger brain is selected for^11–14^. For instance, the Cognitive Buffer Hypothesis (CBH)^15,16^ states that a relatively larger brain allows for increased behavioral flexibility, which facilitates the behavioral buffering of unpredictable changes in the environment^17^. Central to the CBH is that larger-brained species should perform better in more seasonal habitats where cognitive demands are stronger because the available food sources are more difficult to locate in space or time^18,19^. A recent study across birds found support for the CBH in a seasonality context, as brain size and environmental variation showed a positive association^20^. Additional support comes from neotropical parrots in which climatic variability and brain size are positively correlated^21^. Also, non-migrating birds generally have larger brains than migrating ones^22–24^, which is interpreted as a cognitive buffer effect in the residential species; better cognitive performance facilitates performance in a changing/harsh environment, while smaller-brained species evade those challenges via migration. Large brains and seasonality therefore seem to go hand in hand.

However, the brain is among the most energetically costly organs in the vertebrate body^25,26^, environmental factors can therefore impose energetic constraints on its evolution^7,27,28^. Evolution of a larger brain is hence only possible if the supply of energy is increased, if energy is saved by decreasing allocation to another organ (‘trade-off’)^29–33^, or by a combination of the two^27,34^. This energetic perspective on brain evolution is generally termed the expensive brain framework (EBF)^7^. If the EBF is applied to the challenges of seasonality, animals experiencing periodic energy shortages, which are typical for seasonal habitats, may reduce their brain size to endure those periods. Brain size may therefore be negatively correlated with the intensity of seasonality, especially with the duration of periods of low food availability, if they cannot be fully compensated for by increased foraging effort^27,28,35^. This may be analogous to the situations on small islands, where the limited resources are suspected to drive the pattern of relatively smaller brains of mammal species inhabiting those islands^36–39^. The strongest evidence in favor of the EBF stems from strepsirrhine primates in which seasonality was negatively correlated with relative brain size^35^. In the same group of animals, however also the CBH found support^7^. It is therefore unclear whether there is a general relationship between seasonality and brain size evolution across vertebrates. This is especially so as this relationship remains largely unexplored in the poikilothermic vertebrates (fishes, amphibians, reptilians), which comprise >75% of all vertebrate species^40^. The brains of such poikilothermic species should be especially prone to coevolve with environmental/seasonal factors as their scope for metabolic activity completely depends on ambient temperature^41^. Here we therefore provide the first comparative test of the relationship between seasonality and brain evolution in poikilothermic vertebrates, the anurans.

Environmental seasonality (often characterized by variability in mean temperature and/or precipitation, and the length of the dry season, if applicable) often determines food availability; in frogs mostly via influencing the abundance of insects^42,43^. Insect mortality is often determined by the degree of environmental seasonality across species^43–48^, more seasonality therefore results in less food availability. If behavioral flexibility or other cognitive assets help to overcome such periods of food shortage we would expect a positive association between brain size and seasonality (CBH). However, if such periods of food shortage were met by saving energy on overly large brains we would expect a negative association between brain size and seasonality (EBF). We test these opposing predictions in 30 species of Chinese frogs using phylogenetically comparative methods (PGLS; see ‘Methods’) and relate aspects of brain anatomy to three measures of seasonality (Coefficient of variation in precipitation, length of the dry season ‘P2T’, coefficient of variation in temperature; see ‘Material and Methods’ for details).

The CBH and the EBF predict relationships between a species’ whole brain size and the seasonality of its habitat; for brain regions such hypothesis do not exist. However, in birds, the associative areas of the brain (e.g. mesopallium) are known to be involved in learning and hence are the brain regions expected to increase with seasonality^20^. A rich literature on neuro-ecology further shows that brain architecture often reflects the cognitive challenges of a species’ habitat, quality of diet, or predation risk across a number of taxa^9,49–55^. For example, forebrain size is positively correlated with habitat complexity across fishes^50,51^, and the size of the optic tectum is largest in fishes that are piscivorous^3,50^. Similar patterns are found in some prey species, as predation pressure selects for larger olfactory bulbs and optic tectum in anurans^8^ and larger forebrain and optic tectum size in guppies (*Poecilia reticulata*)^56^. In the present study we test the relationship between seasonality and the size of the five main brain regions (olfactory nerves, olfactory bulbs, telencephalon, optic tectum and cerebellum). If we extrapolate the CBH to those regions we predict that seasonality may be positively associated with the size of brain regions that govern behavioral flexibility such as the telencephalon^57^. It is less clear which regions would be expected to change in size with seasonality if energetic limitations play a more prominent role in anuran brain evolution as the EBF postulates.

## Results

**Figure 1:**
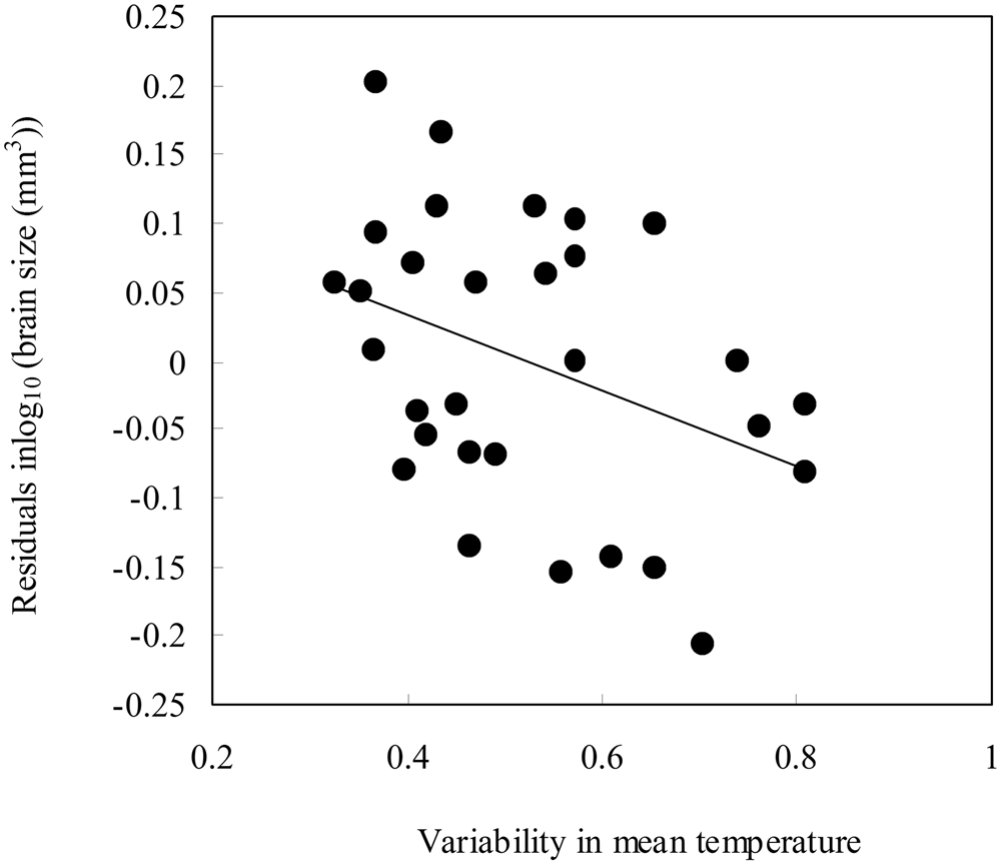
Relationship between relative brain size and variability in mean temperature across 30 anurans species.

Body size, as characterized by snout-vent length (SVL), showed no significant relationship with seasonal variation in temperature (*P* = 0.089), suggesting that in our sample of frog species body size and seasonality show no tight relationship. In the separate models in which we tested the relationships of relative brain size and the degree of environmental seasonality we found a negative correlation between relative brain size and coefficient of variation (CV) in mean temperature when controlling for the effects of phylogenetic relationships, SVL and body mass (Fig. 1; *λ* = 0.126^0.670,<0.001^, *β* = −0.259, *t* = −1.907, *P* = 0.048). In contrast, relative brain size was neither correlated with CV in precipitation (Appendix file: Figure S1; *λ* = 0.234^0.272,<0.001^, *β* = −0.109, *t* = −0.988, *P* = 0.332) nor the length of the dry season (P2T; *λ* = 0.397^0.070,<0.001^, *β* = 0.015, *t* = 1.919, *P* = 0.066).

The multiple linear regressions showed a qualitatively and quantitatively very similar result, as relative brain size was also negatively associated with variability in monthly temperature, but not CV in precipitation and P2T (Table 1). We further found no effects of the CV on temperature among years on relative brain size (*λ* = 0.232^0.189, <0.0001^; *β* = −0.255, *t* = −0.662, *P* = 0.634).

**Table 1:** Regression models of brain size and size of brain regions in relation to various predictor variables for males across 30 species anurans when controlling for phylogeny.

Source	λ	Predictor	β	t	P
*Brain*	0.320^0.266,0.023^	CV in temperature	−0.098	−3.123	0.022
		CV in precipitation	−0.154	−1.292	0.209
		P2T	0.075	0.473	0.640
		Body size	2.134	3.781	<0.001
		Body mass	−0.177	−1.006	0.324
*Olfactory nerves*	0.730^0.051,<0.001^	CV in temperature	−0.106	−1.156	0.260
		CV in precipitation	−0.028	−0.434	0.668
		P2T	−0.003	0.657	0.518
		Rest of brain	0.278	5.838	<0.001
*Olfactory bulbs*	0.713^0.001,0.077^	CV in temperature	−0.080	−1.621	0.118
		CV in precipitation	−0.002	−0.063	0.950
		P2T	−0.001	−0.052	0.959
		Rest of brain	0.138	5.411	<0.001
*Telencephalon*	0.479^0.015,<0.001^	CV in temperature	−0.007	−0.389	0.700
		CV in precipitation	0.011	0.813	0.424
		P2T	0.001	0.683	0.501
		Rest of brain	0.078	8.20	<0.001
*Optic tecta*	0.870^0.149,<0001^	CV in temperature	−0.103	−3.653	0.001
		CV in precipitation	−0.017	−1.486	0.150
		P2T	0.019	1.107	0.278
		Rest of brain	0.094	10.360	<0.001
*Cerebellum*	<0.001^1.0,<0.001^	CV in temperature	−0.021	−0.562	0.579
		CV in precipitation	−0.028	−0.986	0.337
		P2T	<0.001	0.023	0.982
		Rest of brain	0.132	7.058	<0.001

Rest of brain was added as a covariate and was significantly positively related to the size of different brain regions in all models. The partial regression slopes (*β*) for the predictor variable; Phylogenetic signal (*λ)*, *t*- and *P*-values are presented for each model.

**Figure 2:**
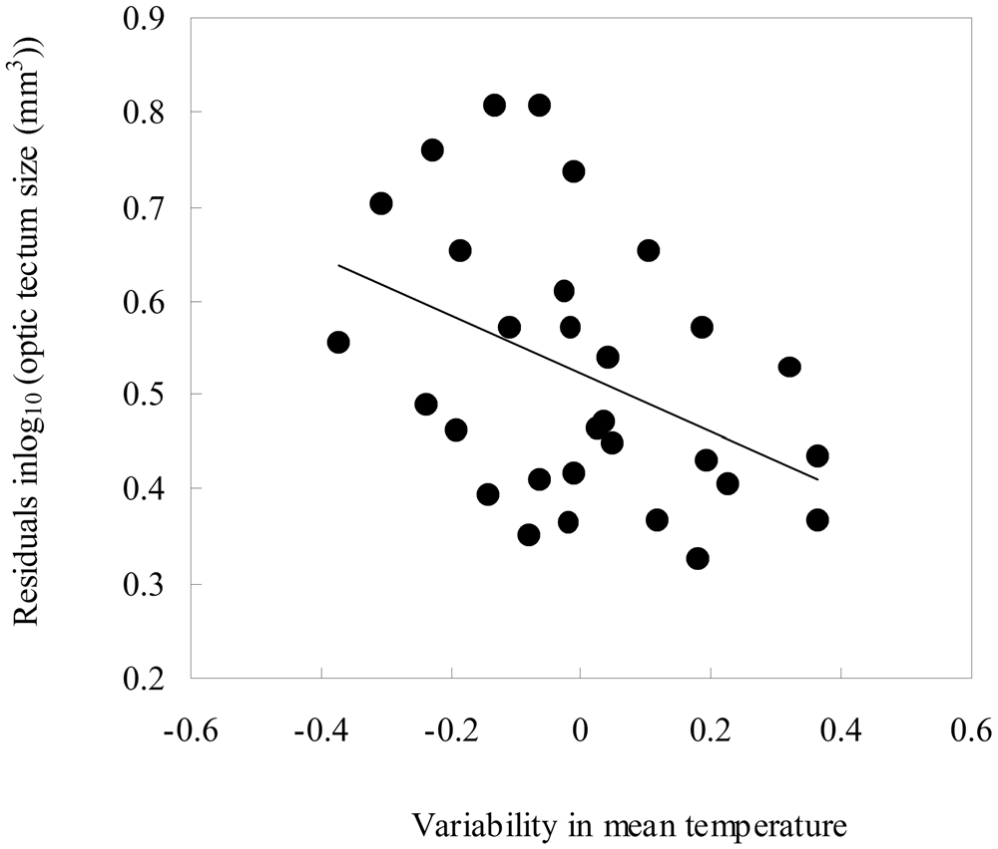
Relationship between relative size of optic tectum and variability in mean temperature across 30 anurans species.

For brain regions, the PGLS controlling for body size revealed that variation in relative size of optic tectum was negatively correlated with variability in monthly temperature (Fig. 2). The fact that we controlled for the effect of decreasing brain size with seasonality (by using brain size as covariate) indicates that frog brains, in addition to getting smaller in overall size, get disproportionally less composed of optic tectum with increasing seasonality. The sizes of all other brain regions (olfactory nerves, olfactory bulbs, telencephalon and cerebellum) were not affected by variability in mean temperature (Table 1). Brain regions sizes were further unrelated to CV in mean precipitation and P2T (Table 1; Appendix file: Figure S2). PGLS revealed that none of the tested brain regions were significantly associated with CV of temperature among years (λ ≤ 0.522^0.001,<0.001^; *β* ≤ 0.224, *t* ≤ 0.546, *P* ≥ 0.342).

## Discussion

Here, we investigated the patterns of brain evolution in the context of seasonality for the first time comparatively in poikilothermic vertebrates. We found a negative association between relative brain size and variability in mean temperature while controlling for possibly confounding variables such as body size and phylogenetic relatedness. Our results are hence consistent with the Expensive Brain Framework. Anurans may tackle recurring food shortages by decreasing brain size in an effort to preserve energy. For brain regions we found only an effect in optic tectum size, which even after the effect of overall brain size was controlled for, was negatively correlated with the variability in mean temperature.

Cognitive buffering of seasonality enables large-brained animals to live in habitats with scare food as exploiting novel and/or changing food sources likely requires behavioral flexibility^7,23^. Seasonality may select for a relatively larger brain, which may be a special case of the CBH^21,58^. In the 30 species of frogs we sampled from areas with considerable variation in seasonality, relative brain size was not associated with variability in mean precipitation and the length of the dry season, indicating that those aspects of seasonality do not impact brain size evolution in anurans. However, we did find a decrease in relative brain size with increasing variability in environmental temperature, which is opposite to what would be expected in a CBH context and instead supports the EBF. This is the first comparative support for the EBF in a seasonality context in non-homoeothermic vertebrates. Currently there is a lack of data showing a clear association between brain size and cognitive abilities in anurans, which is why the CBH should be addressed with caution in anurans (see below). Especially so because Amiel *et al*. (2011) found in amphibians and squamates that successful invaders have relatively larger brains than unsuccessful invaders^59^. Hence, it is too early to conclusively refute the CBH for anurans in a seasonality context.

It is feasible that recurring periods of food scarcity constrain the evolution of brain size because net energy availability is reduced during these times. Anurans exposed to high variability in environmental temperature adapt to a shorter period of climate favorable for foraging, growth and reproduction by increasing the length of hibernation^60^. This may help explain our results, as smaller brains should consume less energy and so allow for longer hibernation periods. However, additional studies are needed to clarify whether hibernation is linked to brain evolution. Importantly our findings corroborate circumstantial evidence from a single species study in *Bufo andrewsi*; these toads occur along a seasonal gradient and specimens collected at different sites revealed a negative relationship between relative brain size and the degree of environmental seasonality^28^. The effect of seasonality on anuran brain evolution is hence evident in both micro-evolutionary process and macro-evolutionary patterns.

The evolutionary response of the brain to the challenges of seasonality seems qualitatively different between poikilothermic and homeothermic animals; birds and mammals seem to largely show a positive association between brain size and seasonality^16,18,20,35^, while the brains of anurans seem negatively associated with seasonality^28^. In any case the benefits of saving energy must outweigh the potential cognitive costs of a decreased brain size, which offers two conceivable explanations to the potential discrepancy between homeothermic and ectothermic species. First, brain size and behavioral flexibility may not be linked in anurans. However, behavioral flexibility and brain size correlates positively in birds and mammals^18–20,35^, and such a relationship may also be inferred in amphibians and squamates^59^. Also the fact that brain size is positively associated with several aspects of cognition in fishes^14,61–64^ and that poison frogs show advanced forms of behavioral flexibility^65^ renders such a scenario unlikely. Second, the brains of ectothermic animals are relatively more expensive than those of homeothermic animals: per unit of mass, ectotherms’ brain tissues consume as much energy as that of homeotherms^25^, but ectotherms have a more than 10-fold lower whole-body metabolic rate^66^. Also, their brain metabolism is less responsive to ambient temperature than that of their bodies^67^. Decreasing investment into brain mass should hence save relatively more energy in ectotherms than homeotherms. We speculate that this relative difference in costliness of brain tissue underlies the opposing evolutionary response of brain size to seasonality in ectotherms versus homeotherms.

We found that the size of most brain regions seemed unaffected by seasonality with exception of the optic lobes, which showed a similar relationship to the variability of mean temperature as overall brain size; the lobes were larger in frogs from stable environments. As selection on whole brain size usually leads to a concerted change in all brain regions^68^, this suggests that a specific selection pressure for visual abilities is in place as it is likely that decreasing optic tectum size concurs with reduced visual abilities^1^. It has been suggested that in anurans a larger optic tectum should allow for better detection of potential predators, which might be an adaptation to high predation risk^8,28^. If seasonality and predator pressure are linked, i.e. there are less predators in habitats with high variability in temperature, it may be possible to decrease optic tectum size while keeping predation risk constant. It is well established that general biodiversity increases with decreasing seasonality^69^, whether this translates into decreased predation pressure with increasing seasonality in anurans remains to be tested. Moreover, the role of the optic tectum in prey localization and capture is a classic topic of neuroethology^8,70–72^. While those studies focus on prey capture it is evident that the optic tectum is responsible for detecting moving objects in the visual field^72^, which may be extrapolated to predator detection Additionally, based on brain morphological comparisons, Taylor *et al*. (1995) suggested a potential trade-off between olfaction and vision across anurans, which may indicate that habitat preference could play a role in anuran sensory system evolution^72^. For instance, if more seasonal habitats would boast a greater number of fossorial (burying) species and a fossorial lifestyle impacts brain anatomy this may impact our results. However, we did not find any indication of uneven distribution of habitat preferences across our samples as fossorial species seemed evenly distributed over the climatic range.

In anuran brain evolution additional aspects may play a role in a seasonality context. Some behavioral features may render anurans more susceptible to the constraints of temperature variability than other vertebrates such as birds, fish or lizards. For example frog movements, including dispersal distances and home range sizes are generally smaller than in other vertebrates^73–75^, frogs may therefore have a decreased ability of leaving unfavorable habitats. In line their habitat preferences are usually highly specific^75,76^, which may imply that the likelihood of the CBH affecting their brain evolution should be low.

Why was relative brain size negatively associated with variability in temperature but not with precipitation or length of the dry season? We may speculate that this relationship is be mediated by insect abundance as the frogs in our sample predominantly feed on insects and other invertebrates^76^ and it is commonly observed that insect biomass depends more strongly on temperature characteristics than on aspects of moisture^43,77,78^. Among other reasons this is thought to be because insects can behaviorally buffer the effects of varying moisture/precipitation by moving towards more favorable microclimates (e.g. by moving deeper into the leaf litter^79^), while temperature effects may be harder to buffer. Hence that temperature, but not moisture aspects were related to brain anatomy is in line with the hypothesis that energetic limitations (in our case low availability of insect prey) underlie the negative association between seasonality and relative brain size. What may speak against this argument is the fact that variability in environmental precipitation has previously been shown to affect abundance of food resource in anurans^42,43^. At this stage more detailed analyses of anuran diet and it’s relationship with seasonality parameters are needed to elucidate the role of food abundance in anuran brain evolution. Especially because previous studies in anurans have shown that environmental variables, such as temperature can be associated with dietary preference^80,81^, because relative brain size and gut size are positively associated^31^, and because gut size sometimes covaries with temperature variability in some populations^80,82^. Integrated studies are therefore needed to fully understand the interplay between diet, seasonality, and gut size in the evolution of anuran brains.

We conclude that our findings are consistent with the EBF and suggest that for anurans variation in temperature can be an energetic challenge that likely constrains brain evolution.

## Materials and Methods

### Ethical approval

All experiments protocols were approved by the Animal Ethics Committee at China West Normal University. All experimental methods were carried out in accordance with the current laws of China concerning animal experimentation, and permission to collect amphibians was received from the ethical committee for animal experiments in China Council on Animal Care (CCAC) guidelines.

### Field sampling

Most frog species exhibit heavily male-biased sex ratios and females are often difficult to obtain^8^, we therefore collected only male individuals. A total of 171 adult male individuals from 30 species of anurans were collected during the breeding seasons 2007–2014 at the Hengduan Mountains in China (Appendix file: Table S1). Each species was sampled in a single site and we recorded the location’s altitude, longitude and latitude as characteristic for each species. All individuals were confirmed to be adult males on the basis of examination of secondary sexual characters^83^, and were transferred to the laboratory and kept in individual rectangular tanks (0.5 m × 0.4 m × 0.4 m) with food for one week^10^. We anesthetized and euthanized them using benzocaine and double-pithing^84,85^. Finally, we preserved all specimens in 4% phosphate buffered formalin for tissue fixation. After two to eight weeks of preservation, we measured the body size (snout-vent length: SVL) to the nearest 0.01 mm with calipers, and body mass to the nearest 0.1 mg with an electronic balance.

Brains were dissected out and weighed to the nearest 0.1 mg using an electronic balance. The number of days samples spent in the buffered formalin prior to dissection does not influence brain mass^31^. We measured total brain volume and the volumes of five major brain regions (olfactory nerves, olfactory bulbs, telencephalon, optic tectum and cerebellum; Table S1). Medulla volume was not determined because pithing damaged the structural integrity of the brain stem. Whole brain mass is not affected by this method^28^. Brain anatomy within a single species can show signs of local adaptation^86–88^. Indeed, when estimating the climatic variation experienced by our frog species across the entire range we found that each species experiences variations in the parameters of seasonality we used (see below for parameter descriptions; all *P* < 0.0001). As each species was collected in a single locality (see below), the potential subtle effects of microevolution and the likely more pronounced cross-species differences are additive in our data set. The strength of this single-location approach lies within the high precision with which climatic variation can be determined for each cohort of animals.

### Brain measurements

All dissections, digital imaging and measurements were performed by two persons (Lou SL & Liao WB) and all measurements were taken blind to the species identity because specimens were coded by uninformative ID-number. We took digital images of the dorsal, ventral, left and right sides of the brain and brain regions using a Motic Images 3.1 digital camera mounted on a Moticam 2006 light microscope at a 400 × magnification (Figure S3 in Appendix file). For dorsal and ventral views, we ensured that the view of the photographed brain was horizontal. We also confirmed that the brain was symmetrically positioned such that one hemisphere did not appear larger than the other. For paired regions, we only measured the width of the right hemisphere and doubled the volume estimate. We measured the length (L), width (W) and height (H) of the brain and the five brain regions from the digital photographs using tpsDig 1.37 software. We defined brain and brain regions as the greatest distance enclosed by the given region and exhibited the used landmarks^28^. Finally, the volumetric estimates of different brains were obtained using an ellipsoid model: volume = (L*W*H) π / (6*1.43)^8^. For all species, there are very high both intra- and inter-measurer repeatabilities of the inter-measurer repeatability for all brain traits^8^. Average size of brain and average size of brain regions for given species were used in all analyses. All variables were log_10_-transformed to meet distributional assumptions before all analyses. To avoid negative values after log transformation, all data were multiplied by 1000 prior to log transformation, as some measurements were smaller than one^89^. The heterogeneity in variability across the five brain regions would not bias the results^8^.

### Phylogeny reconstruction

To reconstruct the phylogeny, we obtained the sequences of six nuclear, three mitochondrial and three mitochondrial ribosome genes from GenBank (for GenBank accession numbers and sequence coverage see Table S2 in Appendix file). The nuclear sequences comprised the recombination-activating gene 1 (RAG1), rhodopsin (RHOD) and tyrosinase (TYR), the mitochondrial genes included cytochrome b (CYTB) and the large and small subunits of the mitochondrial ribosome genes (12 S/16 S). For each locus, we aligned the sequences of all species using multiple sequence alignment (MUSCLE) as implemented in MEGA v.7.0^90^ and determined its best nucleotide substitution model using jModelTest v.2.1.7^91,92^. The best substitution models were GTR + G for 16 S and TYR, HKY + G for RAG1 and RHOD and HKY + G + I for 12 S and CYTB, respectively.

Subsequently, we reconstructed the phylogeny (Appendix file: Figure S4) based on Bayesian inference using Mrbayes V3.2.6^93^. Due to a lack of fossil dates in our sample of species and because the absolute timing of speciation events was deemed less important for our analyses than the relative branch lengths, we omitted the time calibration points. The Markov Chain Monte Carlo (MCMC) simulation was allowed to run for 20 million generations and we sampled a tree every 2000th generation. We used Tracer v.1.6.0^94^ to examine the convergence of the Bayesian chain and the stationary states of all parameters, considering effective sample sizes (ESSs) greater than 200 to be adequate. Finally, we generated a maximum clade credibility tree with mean node heights and a 10% burn-in using Tree Annotator v.1.8.3^95^.

### Data analyses

According to the location of each collected species, we retrieved data on average monthly temperature and precipitation from https://www.meteoblue.com (Table S1)^96^. In this study, we used two standard measures of precipitation variation: i) the coefficient of variation (CV = SD/mean); ii) P2T as a measure of the length of the dry season, a dry month is defined when its total precipitation is less than two times the mean temperature^97^. We also used the coefficient of variation in monthly mean temperature. We also collected everyday average temperature at each location and calculated CV of temperature among years from Chinese Meteorological Stations (http://www.lishi.tianqi.com) between 2011 and 2015. A greater CV of temperature among years may affected brain size, as it might expose animals to higher cognitive demands if they have to behaviorally respond to unexpected changes. Physiologically, anurans can respond to seasonality by increasing the length of hibernation^60^. All collected species hibernate and hibernation length may impact brain evolution. However, it was beyond the scope of the current project to collect data on hibernation length for all species.

The relationships between brain size, size of five brain regions, and the degree of environmental seasonality (i.e. precipitation variation: CV in monthly precipitation and P2T; temperature variation: CV in monthly temperature) were assessed in a series of phylogenetically controlled linear models. To account for the evolutionary relationships among species, we used log_10_-transformed data in the APE-package^98^ in R software package (V.2.13.1)^99^ to perform phylogenetically controlled generalized least-squared (PGLS) regression analyses^100^. According to maximum-likelihood method, the PGLS regression estimates a phylogenetic scaling parameter λ. The parameter λ estimates the effect of phylogenetic signal on the relationships between brain size and environmental factors analyzed (λ = 0 indicating no phylogenetic signal, and λ = 1 indicating strong phylogenetic signal).First we analyzed the relationship between SVL (log-transformed) and seasonal variation in temperature to test whether seasonality is associated with smaller brains or larger bodies. Since brains are subject to a wide range of selective pressures that act simultaneously, we used multiple regressions of phylogenetically controlled linear models treating brain volume as response variable, and environmental seasonality as independent variables, and SVL as covariate to test for relationships between brain volume and the degree of environmental seasonality. To test the relationships between brain regions and degree of environmental seasonality, we used PGLS treating brain regions as response variable (s), environmental seasonality as independent variables, and rest of brain (brain minus respective regions) as covariate. Controlling for potential collinearity between CV in monthly temperature and CV of temperature among years, we used a separate model treating brain regions as response variable, CV of temperature among years as independent variables, and rest of brain as covariate to investigate the effect CV of temperature among years on brain regions.

## Electronic supplementary material

Supplementary Dataset
