# Supplementary material for: Seasonality and brain size are negatively associated in frogs: evidence for the expensive brain framework
Source: Sci Rep. 2017 Nov 30;7:16629. doi: 10.1038/s41598-017-16921-1 (PMC5709389; doi:10.1038/s41598-017-16921-1)

Electronic Supplementary Material

Seasonality and brain size are negatively associated in frogs: evidence for the expensive brain framework

Yi Luo[1,2], Mao Jun Zhong[1,2], Yan Huang[1,2], Feng Li[1,2], Wen Bo Liao[1,2*], Alexander Kotrschal[3]

[1]*Key Laboratory of Southwest China Wildlife Resources Conservation (Ministry of Education), China West Normal University, Nanchong, 637009, Sichuan, China*

[2]*Institute of Eco-adaptation in Amphibians and Reptiles, China West Normal University, Nanchong, 637009, Sichuan, China*

[3]*Zoological Institute, Stockholm University, 10691, Stockholm, Sweden*

*Corresponding author: Wen Bo Liao, E-mail: liaobo_0_0@126.com

Table S1. Species, location, average snout vent length (SVL: mm), body mass (g), brain size (mm^3) and volume of different brain regions (mm^3), CV in temperature, CV in precipitation and P2T.

Species	Latitude	Longitude	SVL	Body mass	Olfactory nerves	Olfactory bulbs	Telencephalon	Optic tecta	Cerebellum	Brain size	CV in temperature	CV in precipitation	P2T
Amolops lifanensis	30°53′	103°00′	52.8 ± 1.6	14.4 ± 0.9	0.54 ± 0.18	0.45 ± 0.07	9.07 ± 1.15	5.29 ± 0.82	0.48 ± 0.08	28.84 ± 3.48	0.74	0.53	0
Amolops loloensis	32°03′	103°54′	52.4 ± 0.8	18.6 ± 1.2	0.27 ± 0.07	1.38 ± 0.18	14.27 ± 0.04	6.82 ± 0.26	0.64 ± 0.04	35.81 ± 1.96	0.65	0.81	4
Amolops mantzorum	30°33′	102°56′	53.8 ± 1.2	14.2 ± 0.4	0.33 ± 0.08	0.89 ± 0.18	10.47 ± 1.41	5.33 ± 0.77	0.39 ± 0.08	29.73 ± 2.30	0.57	0.56	1
Bombina maxima	27º42′	100 º48′	58.1 ± 1.1	19.0 ± 1.2	0.27 ± 0.03	1.05 ± 0.09	13.16 ± 0.78	2.59 ± 0.14	0.27 ± 0.06	23.59 ± 1.25	0.56	0.99	5
Bufo andrewsi	30°33′	102°56′	75.6 ± 1.3	24.4 ± 1.2	0.89 ± 0.11	2.91 ± 0.22	31.41 ± 1.18	6.69 ± 0.38	1.13 ± 0.13	61.55 ± 2.12	0.57	0.56	1
Bufo gargarizans	30°48′	106°06′	99.5 ± 3.6	88.0 ± 1.0	1.29 ± 0.26	4.75 ± 0.41	30.6 ± 2.22	8.87 ± 0.58	0.92 ± 0.08	66.86 ± 3.55	0.39	0.66	6
Bufo melanosctictus	27°23′	108°43′	54.6	20.35	0.44	1.22	13.73	3.26	0.50	25.96	0.49	0.36	0
Bufo minshanicus	32°03′	103°54′	67.5 ± 0.8	36.6 ± 1.6	1.10 ± 0.33	1.57 ± 0.30	12.29 ± 1.62	4.85 ± 0.63	0.66 ± 0.03	30.36 ± 3.01	0.65	0.81	4
Feirana quadranus	32°18′	104°47′	71.1 ± 3.9	43.3 ± 6.6	0.42 ± 0.03	0.43 ± 0.12	12.35 ± 0.67	7.49 ± 1.33	0.48 ± 0.07	33.60 ± 2.17	0.61	0.65	2
Fejervarya limnocharis	28°37′	104°56′	38.1 ± 1.3	5.1 ± 0.5	0.12 ± 0.06	0.68 ± 0.11	7.66 ± 0.55	4.61 ± 0.23	0.26 ± 0.07	21.07 ± 1.23	0.37	0.40	5
Hyla annectans jingdongensis	27°32′	99°18′	33.5 ± 0.9	2.2 ± 0.3	0.01 ± 0.06	0.45 ± 0.04	5.93 ± 0.33	2.18 ± 0.20	0.36 ± 0.02	11.43 ± 0.58	0.81	0.78	2
Hyla tsinlingensis	29°06′	107°08′	32.1 ± 0.5	2.3 ± 0.2	0.05 ± 0.01	0.41 ± 0.03	3.29 ± 0.27	1.39 ± 0.04	0.18 ± 0.04	7.99 ± 0.17	0.70	0.57	0
Hylarana daunchina	27°59′	106°07′	43.7 ± 0.2	9.2 ± 0.2	0.15 ± 0.04	0.48 ± 0.08	6.70 ± 0.31	4.74 ± 0.46	0.29 ± 0.05	19.66 ± 1.13	0.45	0.41	0
Hylarana guentheri	30°50′	106°07′	58.8 ± 0.7	17.5 ± 0.6	1.01 ± 0.10	1.06 ± 0.08	14.24 ± 0.77	10.47 ± 0.49	0.67 ± 0.04	40.49 ± 1.68	0.40	0.67	5
Kaloula verrucosa	26°53′	102°50′	39.2 ± 0.9	6.5 ± 0.4	0.38 ± 0.02	0.77 ± 0.10	9.23 ± 0.79	3.02 ± 0.40	0.35 ± 0.03	20.02 ± 1.22	0.35	0.88	4
Nanorana ventripunctata	27°33′	99°19′	35.5 ± 0.5	4.3 ± 0.9	0.05 ± 0.01	0.19 ± 0.03	6.92 ± 1.35	2.77 ± 0.12	0.16 ± 0.39	14.03 ± 1.10	0.81	0.78	1
Odorrana grahami	27°16′	102°38′	67.6 ± 2.5	27.5 ± 3.4	1.24 ± 0.14	1.66 ± 0.14	18.4 ± 01.31	11.68 ± 0.53	1.02 ± 0.12	55.83 ± 2.28	0.43	0.83	4
Odorrana hejiangensis	28°37′	106°18′	46.6 ± 1.1	7.4 ± 0.5	0.35 ± 0.01	0.65 ± 0.15	7.94 ± 0.85	4.47 ± 0.23	0.33 ± 0.04	20.74 ± 1.53	0.42	0.48	0

Odorrana margaretae	32°40′	106°48′	72.0 ± 0.6	33.2 ± 0.7	0.63 ± 0.19	1.93 ± 0.46	19.96 ± 1.82	16.98 ± 1.24	0.67 ± 0.09	61.82 ± 1.01	0.53	0.69	2
Paa yunnanensis	28°39′	103°22′	60.6 ± 4.8	30.0 ± 4.1	2.29 ± 1.05	1.90 ± 0.10	12.87 ± 2.45	7.05 ± 0.08	1.00 ± 0.18	41.07 ± 7.54	0.47	0.54	0
Pelophylax nigromaculatus	30°50′	106°07′	67.9 ± 2.2	28.3 ± 2.3	0.19 ± 0.01	0.42 ± 0.04	17.71 ± 1.32	6.44 ± 0.57	0.92 ± 0.08	39.70 ± 2.50	0.41	0.67	5
Pelophylax pleuraden	27°11′	102°36′	45.4 ± 0.8	8.4 ± 0.6	0.15 ± 0.08	0.48 ± 0.09	10.12 ± 1.18	6.71 ± 1.08	0.40 ± 0.07	25.76 ± 3.18	0.33	0.83	5
Polypedates megacephalus	29°50′	106°23′	43.6 ± 1.2	5.4 ± 0.4	0.20 ± 0.07	0.44 ± 0.07	10.64 ± 0.81	9.72 ± 0.73	0.87 ± 0.25	30.99 ± 1.91	0.43	0.51	3
Rana chaochiaoensis	27°10′	102°35′	50.4 ± 2.6	9.5 ± 1.3	0.25 ± 0.04	0.74 ± 0.13	6.53 ± 0.55	5.38 ± 0.59	0.31 ± 0.05	22.87 ± 1.53	0.46	0.83	4
Rana zhenhaiensis	27°53′	108°45′	52.7 ± 2.9	14.6 ± 3.8	0.38 ± 0.11	0.71 ± 0.31	8.58 ± 1.71	3.45 ± 1.21	0.50 ± 0.10	21.04 ± 1.89	0.46	0.60	0
Rana omeimontis	28°47′	104°33′	48.0 ± 1.6	11.7 ± 1.4	0.87 ± 0.11	1.28 ± 0.20	13.14 ± 1.42	10.99 ± 1.20	0.77 ± 0.09	39.42 ± 2.74	0.37	0.39	9
Rhacophorus chenfui	28°18′	105°13′	38.6 ± 0.7	5.8 ± 0.7	0.09 ± 0.02	0.60 ± 0.23	7.92 ± 1.00	3.42 ± 0.43	0.26 ± 0.01	17.70 ± 72.96	0.37	0.60	2
Rhacophorus dugritei	28°55′	102°13′	42.2 ± 0.6	5.4 ± 0.2	0.07 ± 0.01	0.31 ± 0.03	10.64 ± 0.40	4.43 ± 0.35	0.45 ± 0.04	23.18 ± 0.83	0.54	0.75	0
Rhacophorus omeimontis	30°33′	102°56′	59.8 ± 1.7	11.6 ± 0.8	0.31 ± 0.04	0.99 ± 0.16	15.81 ± 0.75	9.79 ± 0.79	0.90 ± 0.16	44.69 ± 2.88	0.57	0.56	1
Scutiger muliensis	28°33′	100°56′	62.5 ± 0.40	29.5 ± 3.7	1.13 ± 0.05	1.83 ± 0.02	14.45 ± 2.61	3.98 ± 0.84	0.33 ± 0.09	33.86 ± 5.91	0.76	0.96	4

Table S2. Genbank accession numbers for the gene sequences used to generate the phylogeny.

Species	12S	16S	CYTB	RAG1	RHOD	TYR
Amolops lifanensis	DQ359981	DQ204482	KJ008458	---	DQ360034	DQ360065
Amolops loloensis	AB211455	AB211478	KJ008431	---	DQ360008	DQ360039
Amolops mantzorum	DQ359970	---	KJ008405	EF088240	DQ360023	DQ360054
Bombina maxima	DQ925758	DQ925780	EU531274	---	---	---
Bufo andrewsi	AF160764	AF160782	AF174502	DQ158353	DQ283905	---
Bufo gargarizans	NC_008410	NC_008410	JN647482	KF666177	---	---
Bufo melanostictus	AB167899	AB167927	AF249082	KT031693	AF249097	---
Bufo minshanicus	KM587710	KM587710	---	---	---	---
Feirana quadranus	GQ225906	GQ225932	KX021999	HM163591	EU979886	EU979981
Fejervarya limnocharis	AB277282	AF206466	AB296096	AB526660	DQ458271	EU980027
Hyla annectans jingdongensis	KP742564	KP742693	AY843821	AY844388	AY844574	AY844045
Hyla tsinlingensis	KP742646	KP212702	JX870448	---	---	---
Hylarana daunchina	KU840524	KU840597	KF020631	KU840723	---	KU840782
Hylarana guentheri	AB859216	KF185060	KR264131	KR264365	DQ284009	KR264440
Kaloula verrucosa	KC822507	KC822507	---	---	---	---
Nanorana ventripunctata	EU979717	EU979839	---	HM163585	EU979866	EU979959
Odorrana grahami	EF453731	EU861555	---	EF088257	DQ360016	DQ360047
Odorrana hejiangensis	KU840531	DQ360006	---	KU840727	KU840683	KU840788
Odorrana margaretae	DQ359964	EU861566	KJ815050	EF088261	DQ360017	DQ360048
Paa yunnanensis	GQ225869	GQ225873	KF199150	HM163593	DQ458263	EU979976
Pelophylax nigromaculata	DQ359961	JQ621942	DQ006266	AB360184	DQ283838	DQ360045
Pelophylax pleuraden	JN541324	JQ621943	KR264150	KR264384	DQ360011	DQ360042
Polypedates megacephalus	KU840483	AY880519	AB451722	EU924517	EU924545	KC180271
Rana chaochiaoensis	DQ359975	DQ289107	AF274927	KX269557	DQ360028	DQ360059
Rana omeimontis	DQ289083	DQ289108	AF274928	KX269558	---	KX269785
Rana zhenhaiensis	AB058859	AB058877	JF939105	KX269585	AB728289	KX269811
Rhacophorus chenfui	GQ204763	KU840563	EU924603	EU924519	EU924547	KU840751
Rhacophorus dennysi	DQ019592	DQ019609	EU924604	DQ019512	EU215575	EU924576
Rhacophorus omeimontis	LC010595	LC010595	EU924612	EU924528	EU215565	KU840753
Scutiger muliensis	EF397277	EF397277	---	EF397302	---	---

Figure S1. Relationship between relative brain size and variability in precipitation (a) and P2T (b) across 30 anurans species.

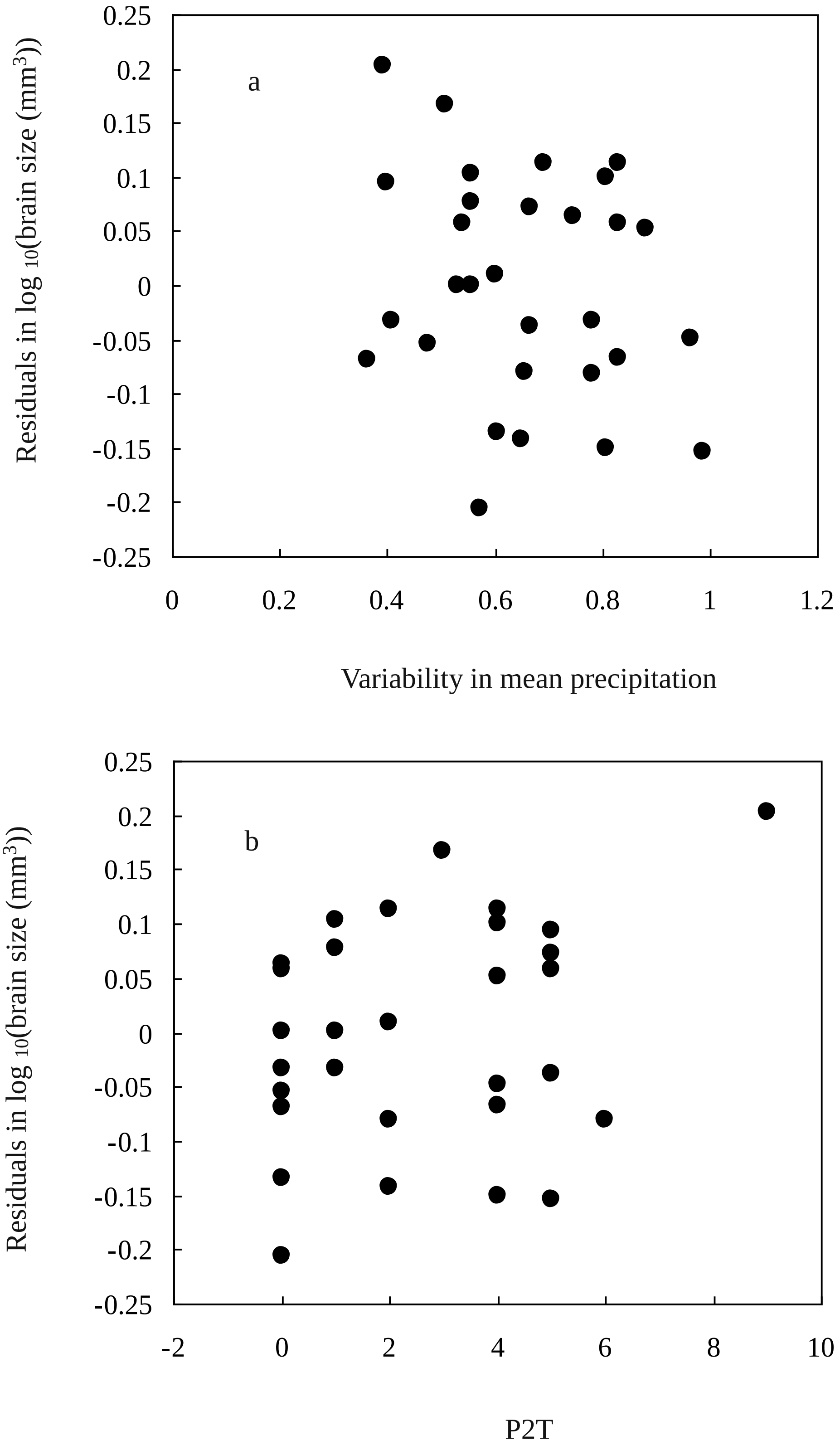

Figure S2. Relationship between relative size of optic tectum and variability in precipitation (a) and P2T (b) across 30 anurans species.

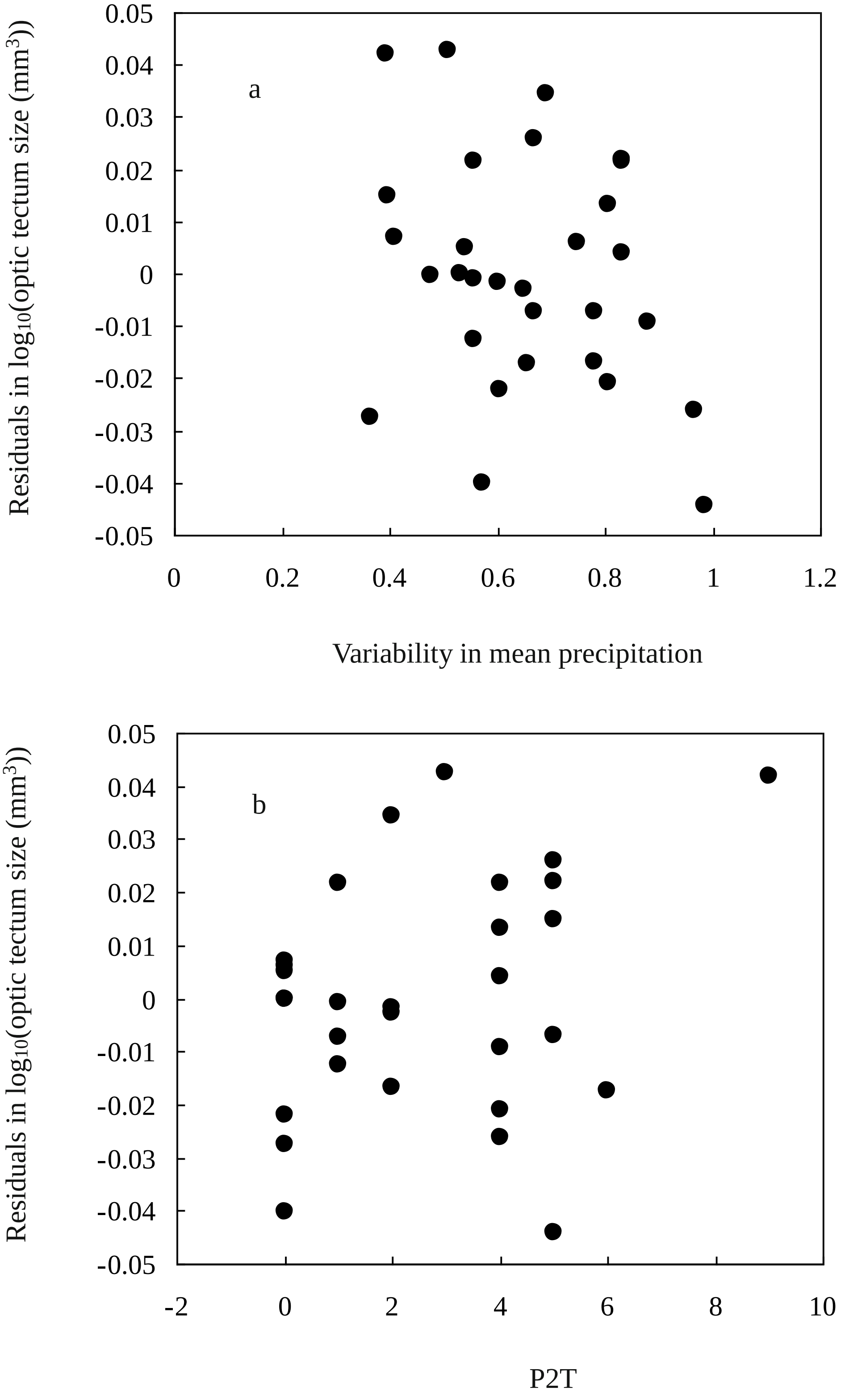

Figure S3. Dorsal, ventral and lateral views of the brain in Anura. Length, width and height measures for each of the five brain parts (olfactory nerves, olfactory bulbs, telencephalon, optic tectum and cerebellum) are shown. See the Methods for further details.

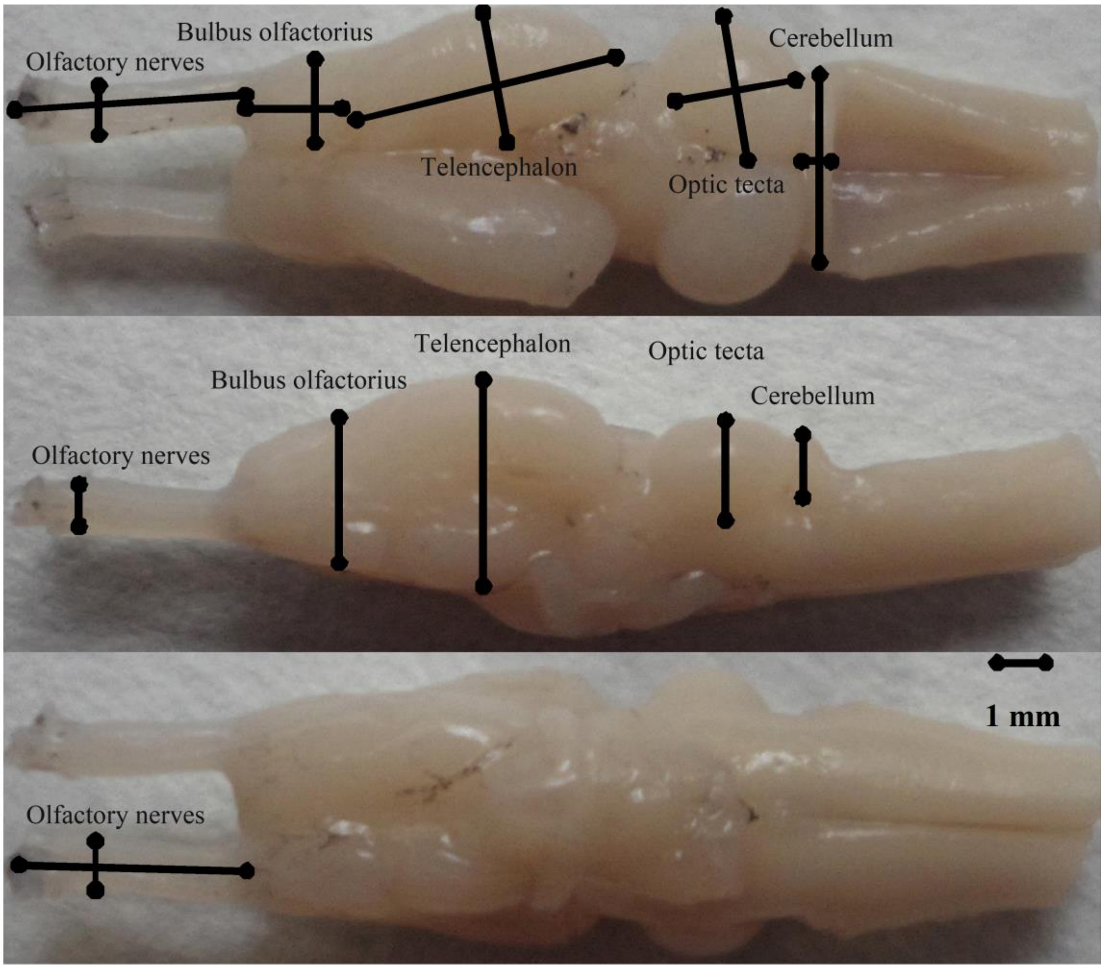

Figure S4. The phylogenetic tree of the 30 anurans species used in the comparative analysis.

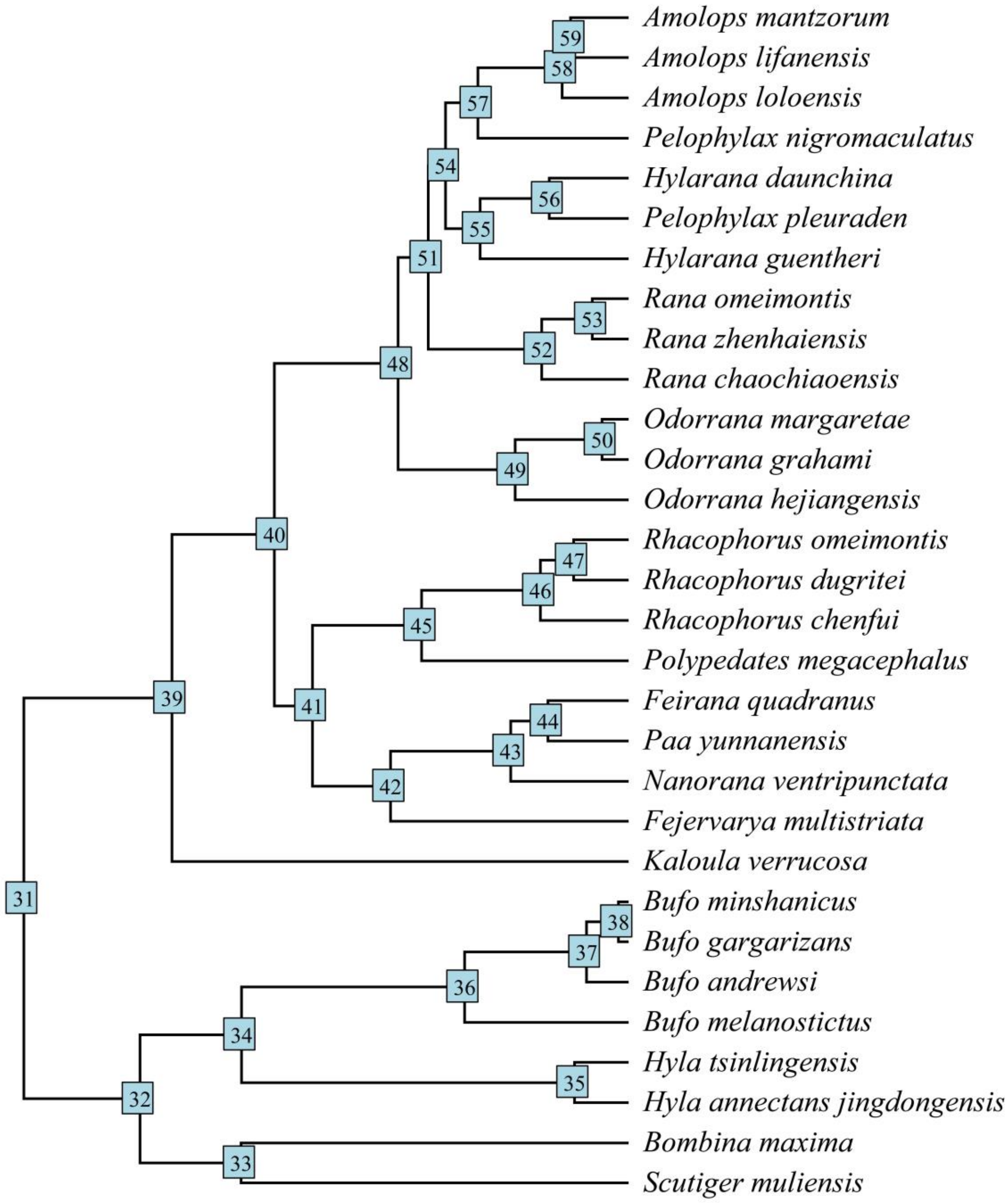

Supplement: Supplementary file 1 — Supplementary Dataset [file 41598_2017_16921_MOESM1_ESM.pdf]
